# Vip3A Resistance Alleles Exist at High Levels in Australian Targets before Release of Cotton Expressing This Toxin

**DOI:** 10.1371/journal.pone.0039192

**Published:** 2012-06-22

**Authors:** Rod J. Mahon, Sharon J. Downes, Bill James

**Affiliations:** 1 Ecosystem Sciences Division, The Commonwealth Scientific and Industrial Research Organisation, Black Mountain Laboratories, Canberra, Australian Capital Territory, Australia; 2 Ecosystem Sciences Division, The Commonwealth Scientific and Industrial Research Organisation, Australian Cotton Research Institute, Narrabri, New South Wales, Australia; Universidad Nacional Autonoma de Mexico, Instituto de Biotecnologia, Mexico

## Abstract

Crops engineered to produce insecticidal crystal (Cry) proteins from the soil bacterium *Bacillus thuringiensis* (Bt) have revolutionised pest control in agriculture. However field-level resistance to Bt has developed in some targets. Utilising novel vegetative insecticidal proteins (Vips), also derived from Bt but genetically distinct from Cry toxins, is a possible solution that biotechnical companies intend to employ. Using data collected over two seasons we determined that, before deployment of Vip-expressing plants in Australia, resistance alleles exist in key targets as polymorphisms at frequencies of 0.027 (n = 273 lines, 95% CI = 0.019–0.038) in *H. armigera* and 0.008 (n = 248 lines, 0.004–0.015) in *H. punctigera*. These frequencies are above mutation rates normally encountered. Homozygous resistant neonates survived doses of Vip3A higher than those estimated in field-grown plants. Fortunately the resistance is largely, if not completely, recessive and does not confer resistance to the Bt toxins Cry1Ac or Cry2Ab already deployed in cotton crops. These later characteristics are favourable for resistance management; however the robustness of Vip3A inclusive varieties will depend on resistance frequencies to the Cry toxins when it is released (anticipated 2016) and the efficacy of Vip3A throughout the season. It is appropriate to pre-emptively screen key targets of Bt crops elsewhere, especially those such as *H. zea* in the USA, which is not only closely related to *H. armigera* but also will be exposed to Vip in several varieties of cotton and corn.

## Introduction

New beneficial traits in populations can arise from selection of existing rare gene polymorphisms (pre-adaptation) or new mutations generated in response to a selection pressure [1]. The extent to which either of these processes contributes to evolution can have different consequences for the rate of adaptation [2]. For instance, mutations of esterase 3 confer malathion resistance on contemporary Australasian *Lucilia cuprina* and PCR analysis of pinned specimens collected before the release of insecticides reveals several cases of changes that conferred resistance. Thus, the early outbreak of resistance in this species can be explained by the pre-existence of mutant alleles encoding malathion resistance [2].

The soil bacterium *Bacillus thuringiensis* produces crystalline inclusions during sporulation which contain insecticidal δ-endotoxins or Cry toxins. Specific *cry* genes engineered into plants to express the proteinaceous toxins, have been the mainstay of transgenic insecticidal crops since the mid-1990’s. In several target pests major gene polymorphisms for resistance to Cry toxins were detected before populations were exposed to Bt crops [3]–[6]. Incipient resistance to Bt was reported in at least one of these targets within several years of exposure to crops [7]. Field level resistance to Bt-crops has been reported in at least 4 target pests [8], [9] but the pre-existing levels of gene polymorphisms for resistance are unknown for these systems.

The usual target pests for Bt crops are Lepidoptera. In general this group are only susceptible to toxins in the Cry1 (e.g., Cry1Ac, Cry1Ab, Cry1F) and Cry2 (e.g., Cry2Ab, Cry2Aa, Cry2Ae) classes, several of which are exploited in existing transgenic crops [8]. Within the Cry1 class, insects which are resistant to one toxin are often, but not inevitably, cross resistant to others [10]. Less is known about cross resistance within the Cry2 class, however Cry2Ab resistant *H. armigera* are resistant to Cry2Aa [11] and Cry2Ab resistant *H. armigera* and *H. punctigera* are resistant to Cry2Ae [12]. Therefore, it is likely for most systems that if resistance emerges to a toxin in the Cry1 or Cry 2 class, there are limited alternative Cry toxins for plant breeders to exploit.

Cry toxins are produced during sporulation [13]. In contrast, vegetative insecticidal proteins (Vips), are secreted during the growth stages before sporulation [14]. Vip proteins are toxic to a taxonomically diverse group of Lepidoptera [14] and show no sequence or structural homology with the δ-endotoxins. Thus, Vip toxins could be efficacious against insects that are resistant to Cry toxins, and provide a third Bt class that could be exploited in transgenic crops. In particular, Vip proteins could be added to crops that also express Cry toxins to produce exceptionally robust pyramids. The value of pyramids stem from the rarity of insects that are phenotypically resistant to more than one toxin. To obtain the full value of any pyramid, the toxins to be ‘stacked’ should require targets to possess separate gene-based mechanisms to overcome each toxin. However the resilience of a pyramid depends on the efficacy of, and levels of pre-existing resistance to, each toxin [15], [16].

In 2009/10 and 2010/11 we used F_2_ screens, which ensure that a proportion of individuals in the second generation of isofemale lines are homozygous for alleles carried by field collected parents [17]. This enabled the establishment of baseline frequencies of alleles conferring resistance to Vip3A in Australian populations of *H. armigera* and *H. punctigera.* Not only did we isolate from field populations the first examples in any insect of alleles conferring resistance to a vegetative insecticidal protein, we also determined that in both target species, baseline frequencies of Vip3A resistance alleles are higher than mutation rates normally encountered. Herein we report for both species the Vip3A resistance allele frequencies, as well as partial characterisation of the isolated resistance, including information on allelism, dominance, genetic basis, and cross-resistance to commercially relevant Cry toxins. We consider the more general consequences of our findings to the potential usefulness of Vip-expressing crops for countering resistance to Bt crops in Lepidopteran pests worldwide.

## Results

For *H. armigera* there was no statistically significant difference between the *r* frequencies obtained for Vip3A in the two study years (2009/10 = 0.029 [11 positive lines, 108 tested lines], 2010/11 = 0.028 [17 positive lines, 165 tested lines]; Fisher’s Exact Test, P = 0.9). Pooling the data across the two study years yields an *r* frequency for *H. armigera* of 0.027 with a 95% CI between 0.019 and 0.038. This frequency is based on 273 tested lines, 28 of which scored positive for resistance to Vip3A.

For *H. punctigera* there was no statistically significant difference between the *r* frequencies obtained for Vip3A in the two study years (2009/10 = 0.008 [5 positive lines, 197 tested lines], 2010/11 = 0.015 [2 positive lines, 51 tested lines]; Fisher’s Exact Test, P = 0.6). Pooling the data across the two study years yields an *r* frequency for *H. punctigera* of 0.008 with a 95% CI between 0.004 and 0.015. This frequency is based on 248 tested lines, 7 of which scored positive for resistance to Vip3A.

Complementation tests involving crosses of the *H. armigera* isolates SP85 and SP477 and the *H. punctigera* isolates Hp8–48 and Hp9–5442 demonstrated that the F_1_ progeny were also resistant to Vip3A (no. larvae at the same stage as parental controls after 7 days/no. larvae tested: *H. armigera*, 44/45; *H. punctigera*, 68/71). This result implies that within each species the resistance in both isolates is due to alleles at a common locus.

Individuals from the SP85, SP477 (*H. armigera*) and Hp8–48 (*H. punctigera*) colonies survived the maximum concentration of Vip3A toxin that could be practically delivered in a surface treatment assay (220 µg/cm^2^) (in all cases, control corrected mortality at this dose was less than 2.4%) and larvae developed at the same rate as siblings reared on non-treated diet. For both species (based on assays with SP85 and Hp8–48), resistance was found to be essentially recessive, with heterozygotes exhibiting concentration-response characteristics that are similar to those of susceptible insects (Table 1). However on 4 out of the 5 occasions tested, the heterozygotes generated from the cross between the resistant (SP85) female and the susceptible (GR) male were more tolerant of Vip3A than the susceptible colony GR or heterozygotes from the reciprocal cross. While this difference was subtle, and generated a resistance ratio of only 3, the difference between the LC_50_ of GR and that of the SP85♀ X GR♂ F_1_’s was statistically significant (X^2^ = 58.51, DF 2, P<0.001), as was the comparison with the reciprocal cross (X^2^ = 51.40, DF 2, P<0.001). In contrast, there was no significant difference in response to Vip3A between GR and the heterozygotes produced by the GR♀ X SP85♂ cross (X^2^ = 0.12, DF 2, P = 0.942). The situation in *H. punctigera* is less clear cut. While heterozygotes were generated on 4 occasions, only the first attempt yielded useful data for both reciprocal crosses (Table 1).

**Table 1:** Susceptibility of various strains of *H. armigera* and *H. punctigera* to Vip3A, Cry2Ab and Cry1Ac.

Species	Vip3A genotype	Colony	Toxin	Slope	LC_50_ ug/cm^2^±95% CI
*H. armigera*	Susceptible (SS)	GR	Vip3A	1.82	0.551±0.37–0.765
			Cry2Ab	1.35	0.030±0.019–0.049
			Cry1Ac	1.42	0.010±0.0064–0.015
	Resistant (RR)	SP477	Vip3A	*	*
			Cry2Ab	1.52	0.0053±0.0033–0.0073
			Cry1Ac	1.64	0.0166±0.0104–0.0254
		SP85	Vip3A	*	*
			Cry2Ab	0.95	0.0077±0.0029–0.0145
			Cry1Ac	2.03	0.0087±0.0059–0.0196
	Heterozygote (RS)	GR♀ X SP85♂	Vip3A	1.92	0.566±0.414–0.736
		SP85♀ X GR♂	Vip3A	2.10	1.473±1.192–1.816
*H. punctigera*	Susceptible (SS)	LHP	Vip3A	1.77	0.594±0.195–0.984
			Cry2Ab	1.38	0.0271±0.0059–0.0588
			Cry1Ac	0.95	0.0362±0.0103–0.0825
	Resistant (RR)	Hp8–48	Vip3A	*	*
			Cry2Ab	2.17	0.0456±0.0022–0.0724
			Cry1Ac	2.29	0.1172±0.0814–0.1601
	Heterozygote (RS)	LHP♀ X Hp8–48♂	Vip3A	1.56	1.340±0.26–2.396
		Hp8–48♀ X LHP♂	Vip3A	1.76	1.382±0.694–2.085

* LC_50_ and slope could not be calculated as there was no increase in mortality at the maximum titre tested (128 ug/cm^2^).

**Figure 1:**
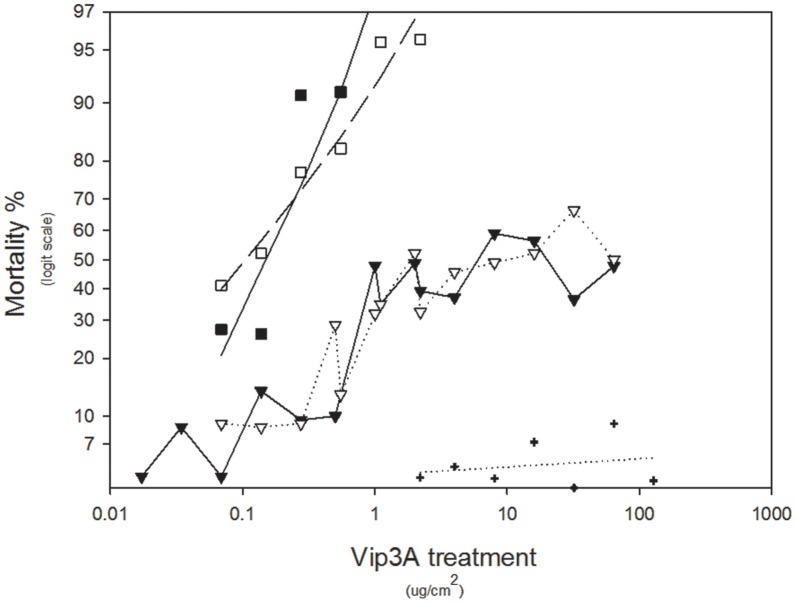
Dose responses of *H. punctigera* genotypes and backcrosses to the resistant colony. Solid squares show data for the homozygous susceptible colony (LHP), open squares show data for the heterozygotes, open triangles show data for the offspring from mating between heterozygous females and resistant males, closed triangles show data for the offspring from mating between heterozygous males and resistant females, and the crosses show data for the homozygous resistant colony (Hp8–48).

**Figure 2:**
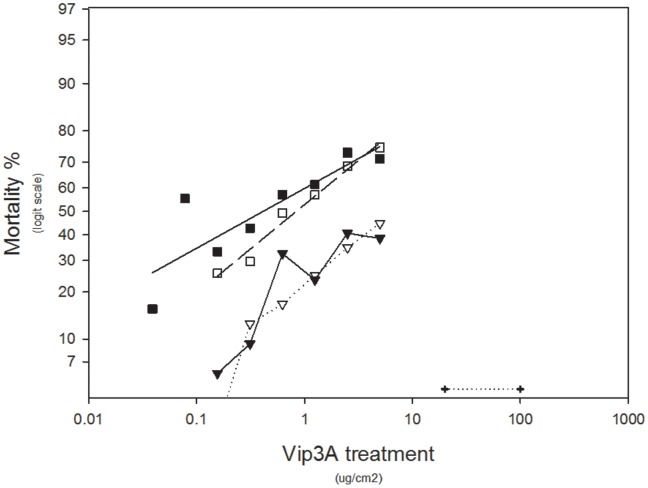
Dose responses of *H. armigera* genotypes and backcrosses to the resistant colony. Solid squares show data for the homozygous susceptible colony (GR), open squares show data for the heterozygotes, open triangles show data for the offspring from mating between heterozygous females and resistant males, closed triangles show data for the offspring from mating between heterozygous males and resistant females, and the crosses show data for the homozygous resistant colony (SP85).

In both species, reciprocal backcrosses of heterozygotes to resistant colonies produced results for concentration-response assays which confirmed that resistance is essentially recessive – that is, 50% of offspring are homozygous resistant while the remainder are heterozygous and thus phenotypically susceptible (see Figures 1 and 2). These data are also consistent with the hypothesis that resistance in both species is conferred by a single gene.

Resistant and susceptible insects were exposed to ranges of concentrations of toxin incorporated into the diet. In two assays, *H. armigera* (SP85) and GR received a maximum of 1200 µg of Vip3A per g of diet. While SP85 were unaffected at this dose (no mortality and 21 of 23 larvae reached 3^rd^ instar by day 7), no GR survived. GR survivors at lower doses grew more slowly with only one of the 20 survivors among the 45 GR exposed to 100 µg toxin per g of diet reached third instar whereas 61 of 64 control larvae reached that stage. Similarly on four occasions the *H. punctigera* resistant colony Hp8–48 and the susceptible HPM colony received a maximum of 800 µg of Vip3A per g of diet. There was no mortality among a total of 86 resistant insects at that dose and 91, 100, 70 and 80% reached third instar by day 7. In contrast, no HPM survived that dose and only 4 of the 23 susceptible *H. punctigera* insects (HPM) exposed to 100 µg per g diet were alive after 7 days and all were first instar. The estimated LC_50_±95% CI for susceptible *H. armigera* and *H. punctigera* was 68.31±35.22–104.25 and 55.173±43.89–70.28 µg per g of diet respectively.

The Vip3A resistant colonies SP85, SP477 (*H. armigera*), Hp8–48 and Hp9–5442 (*H. punctigera*) were established from F_2_ larvae that survived a discriminating-concentration of Vip3A toxin. Additional screens against Cry1Ac or Cry2Ab were conducted with sub-samples of the same cohorts of F_2_ larvae that were exposed to Vip3A; these proved negative suggesting that Vip3A-resistant insects were not cross resistant to additional toxins. This result was confirmed using concentration-response assays where Vip3A resistant insects proved to be as susceptible, or more so, to Cry1Ac and Cry2Ab toxins (Table 1).

## Discussion

Whether in response to recent evidence of emerging resistance to existing toxins [7], [18] or as planned improvements, leading companies that produce Bt crops recently received access to Vip3A toxin [19]. Monsanto’s third generation Bt-cotton, to be traded as ‘Genuity Bollgard III’, will produce Vip3A as well as the Cry1Ac and Cry2Ab toxins present in the second generation dual-gene Bt cotton known as Bollgard II. Genuity Bollgard III is likely to be available in the USA, and begin a phased entry in Australia, around 2016. It will be especially valuable in Australia where Bollgard II is deployed on at least 90% of the cropping area and alleles that confer Cry2Ab resistance in the target pests *H. armigera* and *H. punctigera* occur at frequencies that are of concern [3], [20], [21] and in *H. punctigera* have increased over time [7], [20]. For the USA market, Dow is enhancing Widestrike® to produce Vip3A in addition to the existing Cry1Ac and Cry1F toxins [22]. Bayer plans to licence from Syngenta a Bt-cotton containing Cry1b and Vip3A that trades as Vipcot® [23]. In addition, corn expressing Vip3A and Cry1b (marketed as Agrisure Viptera™) is expected to be available soon [23].

From 2001 to 2003, small trial plots of cotton expressing Vip3A were grown in Australia which proved largely efficacious against Australian *Helicoverpa* spp. [24]. The limited potential exposure of insects to these plots makes it virtually impossible that selection by Bt plants is responsible for the high frequencies of Vip3A resistance alleles that we detected almost 10 years after these trials. We conclude that alleles conferring resistance to Vip3A exist in Australian populations of *H. armigera* and *H. punctigera* as natural polymorphisms. To our knowledge this is the first report of isolation from field populations of alleles conferring resistance to a vegetative insecticidal protein in any insect. Moreover, our data suggest that these pre-existing mutations occur at a relatively high frequency of 0.027 (95% CI, 0.019–0.038) and 0.008 (95% CI, 0.004–0.015) for *H. armigera* and *H. punctigera*, respectively.

The only frequency of resistance to a Bt toxin that is of similar magnitude to the 0.027 for Vip3A alleles in Australian *H. armigera* is the initial estimate of 0.158 for Cry1Ac resistance alleles in *Pectinophora gossypiella* [25]; however, recent estimates are significantly lower at 0.004 [18]. Work on Cry2Ab resistance in *H. armigera* and *H. punctigera* demonstrates that F_2_ screens may underestimate frequencies in natural populations by up to 6-fold [26]. Thus, importantly, the frequency of Vip3A resistance alleles in both Helicoverpa species may be well above the mutation rate of 0.0001 assumed in most models of the evolution of resistance [15]. This suggests that another agent is selecting for an advantage against this toxin (see also [3]) and/or there is a high rate of mutation which introduces resistance alleles that are not selected against [27].

On seeking registration for Vip-producing cotton in the USA, Syngenta cited a maximal concentration of toxin in cotton plant tissues of 23.75 µg Vip3A per g fresh weight [28]. Homozygous resistant neonates of *H. armigera* and *H. punctigera* exposed to concentrations of Vip3A incorporated into diet that were 34–51 times that level (800–1200 µg of Vip3A/g diet respectively) exhibited no increased mortality or reduced growth rate relative to control insects. Cotton tissues contain secondary compounds that are not present in artificial diet, and may interact with Bt toxin to increase its efficacy. For instance, Llewellyn et al. [29] showed that Vip-expressing cotton largely controlled Helicoverpa spp., yet the reported titres of toxin in plants is lower than the LC_50_ we found for susceptible insects in our laboratory assays. In addition, the Vip3A toxin employed here differs subtly from that intended to be used in cotton plants – the sequence of the protein differs by one amino acid and the protein includes a HIS tag. Nevertheless, in our Cry2Ab-resistant *H. armigera* and *H. punctigera* colonies there is a good correlation between the results from diet assays and those using field-grown Cry2Ab cotton plants [S. Downes, unpublished data).

While the frequency of Vip3A resistance alleles in *H. armigera* and *H. punctigera* is uncomfortably high, it is essentially recessive in both species. *H. armigera* heterozygotes created by a cross between male susceptible insects and a homozygous Vip3A resistant female were subtly more tolerant of toxin than susceptible insects. However the difference yields a small resistance ratio of approximately 3. Possible explanations include the presence of a maternal effect, or sex linkage of a gene or genes that subtly modifies the vigour and survival of heterozygotes. Our data demonstrate that Vip3A resistant insects are susceptible to both Cry1Ac and Cry2Ab.

The lack of significant levels of dominance and the absence of cross resistance means that resistance to Vip3A in Helicoverpa should be manageable if Genuity Bollgard III expresses all toxins optimally. Unfortunately, protein levels of Cry1Ac are variable in existing Bt crops, especially later in the season [30], [31]. Llewellyn et al. [29] reported that efficacy of Vip3A declined as the season progressed however it did not do so as markedly as Cry1Ac. Additionally, particularly post flowering, occasional fields of Bollgard II support larvae that are susceptible to Bt and can survive to pupation [32]. Presumably during these episodes the expression of toxin(s) declines, and since Cry2Ab expression is more stable than Cry1Ac [33] there are likely to be periods where only Cry2Ab is effective, thereby selecting for resistance (see [32], [34]). Thus there is considerable merit in protecting the susceptibility of Helicoverpa spp. to Cry2Ab until Bollgard III becomes available, otherwise Vip3A may be exposed to selection in a similar fashion to what we assume currently occurs for Cry2Ab in Bollgard II.

As we have no knowledge of the evolutionary forces maintaining Cry2Ab or Vip3A resistance polymorphisms in populations prior to the release of Bt-crops expressing these proteins, it is difficult to predict their frequency in pests elsewhere in the world. It is possible that the environmental-ecological conditions needed to generate or maintain these polymorphisms occur only in Australia. *H. armigera* and *H. punctigera* are only distantly related, yet both exhibit evidence of such polymorphisms prior to use of the toxins in Bt plants. Either the environmental-ecological conditions needed to generate or maintain these polymorphisms occur only in Australia or, such polymorphisisms are likely to be encountered in other Helicoverpa pests. Furthermore, the New World congener *H. zea* is very closely related to *H. armigera*, thus resistance to Vip3A in this species is of special interest.

Vip-expressing crops may soon be grown widely. It may be wise for organisations responsible for developing resistance management programs for Bt toxins, particularly those targeting species of Helicoverpa, to survey for Vip3A resistance alleles prior to the widespread use of this toxin. While targeted pest species will be naive to the Vip3A present in plants, they, like Australian Helicoverpa, may already possess the genetic variability to quickly respond to this new agent.

## Materials and Methods

A Vip3A clone in *E. coli* was used as a source of toxin. The methods employed to produce the Vip3Aa toxin generally followed those described in [35]. The *vip3A* gene was modified to contain a HIS tag sequence at the C terminus of the protein to facilitate purification. *E. coli* cells were grown at 37°C in a shaking incubator overnight in Luria-Bertani medium. Expression of Vip3A protein was induced by the addition of isopropyl-D-thiogalactopyranoside (IPTG) to a final concentration of 1 mM at O.D._600_ of 1.2 and cells were further cultivated at 28°C overnight. Cells were harvested by centrifugation for 15 minutes at 5000 g and then resuspended and sonicated in PBS (137 mM NaCl, 2.7 mM KCl, 4.3 mM Na_2_HPO_4_, 1.5 mM KH_2_PO_4,_ pH 7.4) before being partitioned into aliquots and frozen. Purified Vip3A was prepared from a portion of the sonicated cell lysate using a HIS SELECT 1 ml cartridge (Sigma) and was examined for purity and stability by sodium dodecyl sulfate-polyacrylamide gel electrophoresis (SDS-PAGE) analysis. Protein concentration of the purified sample was quantified by using the Bradford method with BSA as a standard. Parallel surface contamination bioassays using either purified toxin or sonicated cell lysate were performed to calibrate the toxicity of the crude solution which was used as a source of toxin in all further assays.

Response to increasing doses of Vip3A toxin were examined in a Bt-susceptible colony of *H. armigera* (designated ‘GR’) and *H. punctigera* (designated ‘HPM’) to establish appropriate concentrations to identify likely instances of resistance in F_2_ tests. We have found that the susceptibility of the two colonies is typical of the vast majority of field collected insects (S. Downes, unpublished data). Based on these results, discriminating assays were established for the two species. For *H. armigera,* 10 µg/cm^2^ of toxin was applied to the surface of cooled diet while for *H. punctigera* a lower application rate of 2 µg/cm^2^ was employed.

To detect Vip3A resistance, we employed the F_2_ techniques previously described for *H. armigera* and *H. punctigera* to screen against Cry1Ac and Cry2Ab [4], [11]. Indeed, the only change to our regular routine was to expose an additional sample of approximately 90, F_2_ larvae to Vip3A toxin whereas previously we tested only for the two Cry toxins. We followed Baysian inference statistical approaches [17] to determine expected frequency of resistance alleles in the sampled populations (E[*p_R_*]) and the 95% credibility intervals for our estimated frequencies.

To examine the characteristics of Vip3A resistance, we studied the *H. punctigera* Vip-resistant colony Hp8–48 which was isolated during the summer of 2008–09 from individuals collected as eggs on cotton in the Gwydir valley. To perform complementation assays we also used the *H. punctigera* Vip-resistant colony Hp9–5442 which was isolated during the summer of 2009–10 from individuals collected as eggs on pigeon pea in the Macintyre valley. To determine the characteristics of Vip3A resistance in *H. armigera,* we examined the Vip-resistant colonies, SP85 and SP477 which were isolated during the summer of 2009–10 from individuals collected as eggs on non-Bt cotton from St. George, Qld (SP85) and the Gwydir Valley, NSW (SP477).

The majority of tests involved surface treatment assays as described in [11] and were repeated at least 3 times using different cohorts of the same colony. Briefly, the surface of aliquots of cooled diet in wells of a 45-well plastic tray was contaminated with a solution of toxin and allowed to air dry. When dry, an individual neonate was added to each well and sealed in place with a perforated lid. After incubation at 26°C for 7 days, assays were scored for ‘dead’ (incapable of coordinated movement) and live insects, and the instar was recorded for the latter. Such assays were used to conduct complementation tests by exposing to toxin the offspring of reciprocal crosses between (a) the SP85 and SP477 *H. armigera* strains, and (b) the Hp8–48 and 9–5442 *H. punctigera* strains. We also used these assays to examine possible dominance present among neonate heterozygotes produced by crossing the resistant and susceptible strains. Finally, surface treatment assays were used to examine the response of the Vip3A resistant strains to Cry toxins Cry2Ab and Cry1Ac.

We also used diet incorporation assays to further investigate the extent of resistance to Vip3A by the resistant strains. Serial doses of Vip3A toxin were mixed with diet cooled to 45°C, and then poured into 100 mm petri dishes. Once cooled, portions of diet approximately 6 mm^3^ were placed in wells of our standard 45-well assay plates. One neonate was placed in each well which was then heat sealed.

No specific permits were required to collect the insect material used in the described research program from commercial farms. In all cases, permission to access and collect insects from commercial fields was obtained directly from the landowners. None of the accessed commercial farms were protected in any way, and our field studies did not involve endangered or protected species.
